# Management of *Varicella* Gangrenosa: A Life-Threatening Condition from Chickenpox

**DOI:** 10.1155/2014/206152

**Published:** 2014-11-19

**Authors:** Judith P. M. Schots, Peter Moons, Jan H. M. B. Stoot

**Affiliations:** ^1^Department of Surgery, Orbis Medical Centre, P.O. Box 5500, 6130 MB Sittard, The Netherlands; ^2^Department of Surgery, Atrium Medical Centre, Heerlen, The Netherlands; ^3^Department of Pediatrics, Haga Hospital, The Hague, The Netherlands

## Abstract

*Varicella* gangrenosa, in which gangrenous ulceration of the skin and/or deeper tissues is seen, is a rare but alarming complication of *Varicella* infection. An early surgical intervention is generally advised, especially in case of sepsis and/or the presence of large necrotic lesions. We describe a case of a previously healthy 12-month-old boy presenting with sepsis due to *Varicella* gangrenosa. He presented with moderate lesions of moist gangrene. We treated our patient initially with antibiotics (ceftriaxone and metronidazole) and later on flucloxacillin and antiviral therapy (acyclovir) whereupon his condition rapidly improved and all skin lesions healed entirely. This report highlights the possibility of conservative treatment and emphasizes the significance of acyclovir in the management of chickenpox complicated by moist gangrene due to bacterial superinfection.

## 1. Introduction

Chickenpox (*Varicella*) is a very common disease in childhood that results from primary infection with* Varicella zoster virus*. It starts with the appearance of a characteristic papular exanthema and symptoms including mild fever, malaise, nausea, and headache.* Varicella* usually has a benign course. However, complications may occur and can be life-threatening causing sepsis, osteomyelitis, myocarditis, and encephalitis. Dermatological complications are superficial bacterial superinfection, sometimes leading to necrotizing fasciitis, hemorrhagic chickenpox, and* Varicella* gangrenosa. Although* Varicella* complications were previously thought to depend on the immune status, a relationship of* Varicella* gangrenosa and an immunological disorder has not been reported. Healthy, immunocompetent children and adolescents may experience complications related to* Varicella* infection as well. We describe a previously healthy 12-month-old boy presenting with moist gangrene due to* Varicella* infection. Furthermore, we provide an overview of current evidence concerning the treatment of* Varicella* gangrenosa.

## 2. Case Report

**Figure 1:**
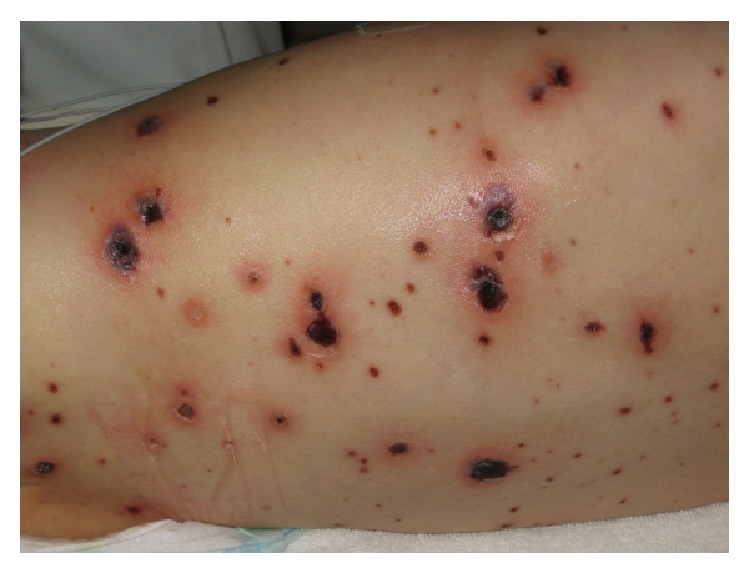
Varicelliform rash with necrotic lesions on day one on the back of our patient.

**Figure 2:**
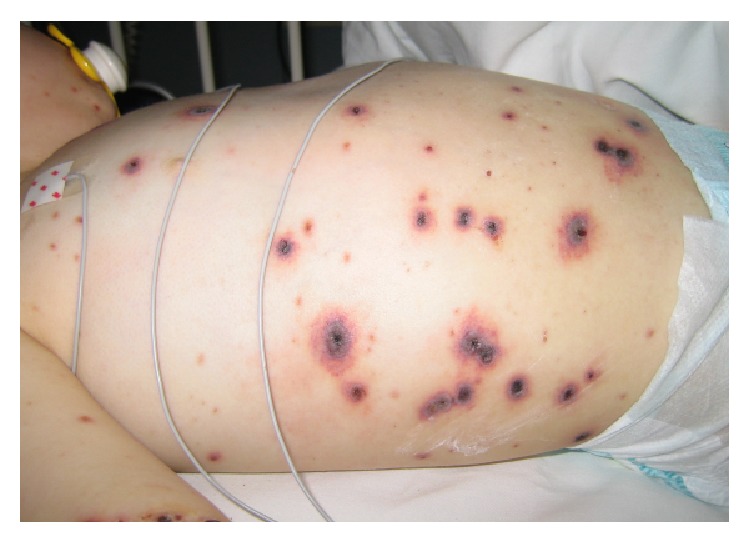
The abdomen appeared tender with edema and necrotic lesions with a maximum diameter of 3 centimeters.

A previously healthy 12-month-old boy was admitted five days after he developed the typical rash of chickenpox. He was referred to our hospital because of high fever and skin necrosis on some of the lesions. On examination in the hospital he appeared ill and mildly irritable; his temperature was 39°C with normal vital signs. A varicelliform rash was seen on the scalp, trunk, and extremities. Several necrotic lesions (maximum diameter of 3 cm) were predominantly present on the back and abdomen (Figures 1 and 2). Results of the laboratory tests showed leukocytosis (28.8 × 10^9^/L) and elevated C-reactive protein level (153 mmol/L). Coagulation studies and lumbar puncture were normal. An abdominal X-ray showed pneumatosis intestinalis; abdominal ultrasound was normal. He was initially given broad spectrum antibiotics, ceftriaxone and metronidazole. Our patient was stable until, on the third day of admission, new lesions appeared. In a culture of the fluid from one of these lesions* Varicella zoster virus* and* Staphylococcus aureus* were found. Cultures of blood and cerebral spinal fluid were negative. T-cell subpopulation and T-cell stimulation tests did not show signs of cellular immunodeficiency. Antibiotic regimen was changed to flucloxacillin. Acyclovir was started whereupon skin lesions immediately started to heal and fever diminished. His condition rapidly improved and our patient was discharged after eleven days of hospitalization. All skin lesions healed entirely and up till now, he is doing excellently.

## 3. Discussion

*Varicella* gangrenosa, in which gangrenous ulceration of the skin and/or deeper tissues is seen, is a rare complication. The exact incidence of* Varicella* gangrenosa is unknown. Three types of* Varicella* gangrenosa have been described in literature: (A) moist gangrene, which is thought to be infective, most often caused by* hemolytic Streptococcus* or* Staphylococcus aureus*; (B) dry gangrene, secondary to arterial thrombosis; (C) purpura fulminans, associated with disseminated intravascular coagulation (DIC) [1]. It is of utmost importance to differentiate between the subtypes of* Varicella* gangrenosa and other skin infections, while these conditions warrant alternative therapy.

Moist gangrene is characterized by erythema which changes within 24–48 hours into blue, wet lesions [2]. Lesions can appear as small spots arranged diffusely on the body and progress rapidly into bigger crusted lesions. It can be difficult to distinguish moist gangrene from other skin infections. However, there are some little differences that might be clues in the process of diagnosing. Dry gangrene generally involves the tips of fingers or toes and is characterized by cool, dry, discolored lesions [1]. Purpura fulminans is characterized by thrombocytopenia and bleeding of the mucous membranes and gastrointestinal tract [2, 3]. Necrotizing fasciitis is characterized by red/violet, swollen skin with crepitus and blisters may form. The most typical feature is pain out of proportion to the physical findings [4]. There is no evidence that laboratory tests or imaging techniques (CT and MRI) are useful in establishing the diagnosis, in decreasing neither the morbidity nor mortality of these skin infections [4].

Currently the best treatment option is debatable. In the authors opinion, in the presence of gangrene associated with DIC, gangrene secondary to arterial thrombosis, or necrotizing fasciitis, aggressive surgical debridement is the treatment of first choice. However, our patient presented with moderate lesions of moist gangrene and was treated successfully with antibiotics and antiviral therapy. Although differentiation between the subtypes of gangrene might be difficult, we believe conservative treatment must be considered. Conservative treatment should emphasize on close monitoring of clinical signs, fluid resuscitation, and administration of *β*-lactam antibiotics (penicillin) and acyclovir [2, 3, 5]. Acyclovir is one of the three approved antiviral therapies for* Varicella* infection in the United States. The American Academy of Pediatrics strongly recommends treatment with acyclovir within 24 hours of rash onset for children with underlying immunodeficiency, or certain groups at high risk of severe* Varicella* or its complications [6]. In The Netherlands antiviral therapy is recommended in high risk patients with* Varicella* complications [7]. However, usage of acyclovir in immunocompetent children experiencing* Varicella* with complications remains uncertain. In the early 1990s, Balfour et al. used acyclovir for treating 50 out of 102 otherwise healthy children diagnosed with* Varicella* [8]. The other 52 children received placebo. Acyclovir recipients showed a significant reduction of duration and severity of fever and a significant reduction of the amount of lesions, compared with the placebo group, when acyclovir was initiated within 24 hours of rash onset. Moreover they experienced an accelerated cutaneous healing, without any adverse effects. A recent Cochrane review conducted by Klassen et al. showed comparable results; however the number of days to no new lesions and relief of itchiness did not reduce significantly [9].

We started acyclovir when new vesicles appeared and our patient rapidly improved afterwards. We are opposed to routine usage of acyclovir in immunocompetent children diagnosed with* Varicella* without any complication. However, we believe that acyclovir might be added in the therapeutic regimen when* Varicella* is complicated by moist gangrene, to reduce the duration and severity of fever, to accelerate cutaneous healing, and to decrease the risk of developing other severe complications. We advise initiating treatment at a dose of 800 mg orally, five times a day for 7 days. In case of complicated* Varicella*, acyclovir dosage should be increased to 10 mg/kg IV for 7–10 days, to reduce the incidence of complications [5].

In conclusion, in case of a patient with moderate lesions of moist gangrene, conservative treatment must be taken into consideration, while successful conservative management is possible. Furthermore, the early administration of acyclovir, preferable within 24 hours after rash onset or within 72 hours after new vesicle formation, may be beneficial in the management of* Varicella zoster virus* complicated by moist gangrene in otherwise healthy children.
